# Comparative micromorphology and anatomy of flowers and floral secretory structures in two *Viburnum* species

**DOI:** 10.1007/s00709-016-0972-0

**Published:** 2016-04-13

**Authors:** Agata Konarska

**Affiliations:** Department of Botany, Faculty of Horticulture and Landscape Architecture, University of Life Sciences in Lublin, Akademicka 15, 20-950 Lublin, Poland

**Keywords:** Histochemistry, Micromorphology and anatomy, Nectaries and trichomes, Stigma microstructure, *Viburnum* flowering

## Abstract

In entomogamous plants, the presence and function of floral secretory structures, whose main role is to attract pollinators, is strictly associated with the pollination ecology and hence the reproductive success of the plant. The aims of the present paper were to analyse the micromorphology and anatomy of flower nectaries and stigmas in *Viburnum opulus* and *V. lantana* and to determine the function and microstructure of inflorescence trichomes in both taxa using light and scanning electron microscopy as well as histochemical assays. It was found that stigmas were formed by papillae, which contained lipids, polysaccharides, tannins, and pigments. Stigmatic secretion proceeded via cuticular pores. Floral nectaries formed a thick layer around the styles, and nectar was secreted through numerous nectarostomata. There were no traces of vascular bundles penetrating the nectary tissue. In turn, numerous tannin deposits were observed in the cells of the glandular parenchyma. Pedicels, hypanthia, and bracts had mainly peltate and capitate glandular trichomes as well as stellate non-glandular trichomes (in *V. lantana*). The trichomes were shown to contain lipids, mucilage, and tannins. Many similarities in the flower and nectaries microstructure and considerable heterogeneity were observed in the examined *Viburnum* species. Knowledge of the microstructural characteristics of flowers, nectaries, and trichomes may be important for the phylogenesis and taxonomy of the genus *Viburnum* and the family Adoxaceae. Additionally, floral and nectaries features are helpful in assessment of the relatedness between taxa and provide better understanding of the floral biology and pollination ecology.

## Introduction

The genus *Viburnum* L. representing the family Adoxaceae comprises approximately 160–200 species of evergreen, semi-evergreen, and deciduous shrubs or small trees growing primarily in the temperate climate zone of the northern hemisphere and in tropical mountains, south-eastern Australia and Tasmania, with their largest diversity in Himalaya and China (Winkworth and Donoghue 2005). Besides *Viburnum*, i.e. the most numerous genera in Adoxaceae, the family comprises only a few representatives: *Adoxa* L., *Sinadoxa* C. Y. Wu et al., *Tetradoxa* C. Y. Wu., and *Sambucus* L. (APG Angiosperm Phylogeny Group 2009). *Viburnum* differs markedly from the rest of the family in several aspects, e.g. in having simple, rarely ternate leaves, introrse anthers, single-seeded drupes, and scalariform vessel perforations (Donoghue 1983a, b; Winkworth and Donoghue 2004, 2005). Furthermore, the development and structure of the ovary, abortion of two of the three carpels, and the fruit morphology and anatomy distinguish *Viburnum* among other Adoxaceae representatives (Wilkinson 1948). There are also differences in the location and type of floral nectaries. In *Adoxa*, *Sinadoxa* and *Tetradoxa*, multicellular trichome nectaries are located at corolla lobe bases; in contrast, many *Sambucus* species lack nectaries, whilst in others (*S. javanica*, *S. chinensis*) whole sterile flowers in the inflorescence were converted into nectaries (tzw. substitutive nectaries) (Vogel 1997; Donoghue et al. 2003). In turn, the floral nectaries in the genus *Viburnum* are located at the apex of the gynoecium, forming an ovary-roof nectary (Erbar 1994; Erbar and Leins 2010; Tank and Donoghue 2010).

In terms of taxonomy, the genus *Viburnum* is a very difficult group due to the high possibility of hybridization (Lobstein et al. 2003). The patterns of diversification in *Viburnum* have been investigated by Moore and Donoghue (2007, 2009) and Clement et al. (2014). Earlier descriptions of the taxonomic differences between *Viburnum* species were primarily focused on the presence of extrafloral nectaries (Weber et al. 2012; Clement et al. 2014), form of the inflorescence (Jin et al. 2010; Tank and Donoghue 2010), flower morphology and vascularisation (Wilkinson 1948), structure of the pollen grain exine (Donoghue 1985; Maciejewska 1997), as well as the density and type of trichomes (Clement and Donoghue 2011; Prabhu and Ponnudurai 2011; Clement et al. 2014).

The numerous, tiny *Viburnum* flowers form corymb-like or panicle-like inflorescences with a diameter of 5–15 cm and composed of 15 to over 500 hermaphroditic flowers; in some species, they also have a fringe of large sterile flowers around the perimeter of the inflorescences acting as a pollinator target (Donoghue 1980; Jin et al. 2010). The androecium is composed of five stamens with filament bases attached to corolla petals; in turn, the gynoecium has 3 connate carpels with inferior ovaries, two of which are aborted. The style is short, slightly three-lobed, and fairly broad (Donoghue 1980; Donoghue et al. 2003). As demonstrated by Jin et al. (2007), viburnums are self-incompatible and seed production depends on pollinator visits.

Since the current knowledge of the structure and function of flower nectaries and other floral secretory structures is insufficient, the aim of the present paper was to show for the first time the micromorphological and anatomical organization of the stigmas and floral nectaries in *Viburnum opulus* L. and *V. lantana* L. and to determine the distribution, structure, and role of glandular trichomes located in the proximity to the flowers in both taxa. *V. opulus* L. and *V. lantana* L., characterised by medicinal properties and ornamental values, are common species across Europe values (Altun et al. 2008; Dirr 2009). The species differ considerably in the morphological traits of their flowers (Table 1 and references therein). Comparative studies of the microstructural traits of stigmas, nectaries, and trichomes in the *Viburnum* genus, which comprises many sometimes only slightly differing species, may be important for the phylogenesis of this taxon (Winkworth and Donoghue 2005; Clement and Donoghue 2011; Weber et al. 2012). Moreover, as shown by many authors, the location and structure of nectaries (Galetto and Bernardello 2004; Zini et al. 2014; Konarska 2015) and trichomes (Behnke 1984; Harder and Barrett 2006) are important taxonomic characteristics helping in assessment of the relatedness between taxa and facilitating better understanding of the floral biology and interaction between the plant and the pollinator (Anderson 1995; López and Galetto 2002).

**Table 1:** Characteristics of *V. lantana* and *V. opulus* inflorescences and flowers (Donoghue 1980; Kollmann and Grubb 2002; Tank and Donoghue 2010)

Characteristics	*V. opulus*	*V. lanatana*
Flowering time	From June to July	From late April to June
Inflorescences	Umbel-like, with a glabrous or short glandular peduncles	Umbel-like with a hairy peduncles
Flower buds and bracts	Buds with short pedicels and with one to two pairs of bracts, red-brown, usually glabrous	Buds at least partly covered by two small hairy bracts
Colour and shape of flowers	Flowers yellowish-white, bell-shaped	Corolla creamy-white, rotate, funnel-shaped
Form, smell, and size of flowers	Flowers of unequal size, differentiated into much larger outer sterile flowers and inner hermaphroditic, same-size, fertile flowers with an unpleasant, sickly smell	All flowers fertile, with a similar size and form, and with an unpleasant smell

## Materials and methods

The *Viburnum lantana* L. (wayfaring tree) and *V. opulus* L. (guelder rose) shrubs originated from the Arboretum of the UMCS Botanical Garden in Lublin, SE Poland (51°15′44′ N, 22°30′48′ E). In 2015, the full bloom was noted in early May for *V. lantana* and in the third decade of May for *V. opulus*. Freshly opened, nectar-secreting flowers of the two taxa sampled together with the pedicels and bracts were analysed under a light stereoscopic microscope Olympus SZX12 and a bright field light microscope, and after fixation, under a scanning electron (SEM) and light (bright field and fluorescence) microscopes (LM).

For SEM observations, samples of flowers were fixed in 2.5 % glutaraldehyde in phosphate buffer (pH 7.4, 0.1 M) at 4 °C for 12 h. Then, the material was washed in phosphate buffer and dehydrated in a graded acetone series. The plant material was subsequently subjected to critical point drying using liquid CO_2_, sputter-coated with gold, and examined at an accelerating voltage of 30 kV using a TESCAN/VEGA LMU scanning electron microscope. The measurements of the styles, stigmas, and nectaries (*n* = 10) as well as nectarostomata (*n* = 20) were taken using morphology software coupled with SEM. The surface area of the nectaries was calculated according to the formula for the surface area of a truncated cone.

To obtain semi-thin sections, flower fragments were fixed in 2.5 % glutaraldehyde in 0.1 M phosphate buffer at pH 7.2 for 12 h at 4 °C temperature. Then, they were carefully washed three times in phosphate buffer and dehydrated in an ethanol series and embedded in LR white resin (LR white acrylic resin, medium grade, Sigma-Aldrich). Semi-thin sections, in thickness from 0.7 μm, were cut with glass knives using a Reichert Ultracut S ultramicrotome. For general histology, semi-thin sections were stained with 1 % aqueous methylene blue-azure II solution (O’Brien and McCully 1981). The presence of water-insoluble polysaccharides was tested using Periodic acid-Schiff’s (PAS) reagent (O’Brien and McCully 1981) after blocking of free aldehyde groups. The sections were also examined by means of fluorescence microscopy for detection of cutinized cell walls and also for the presence of lipids. For this purpose, the semi-thin sections were examined using a filter set, green FITC (EX 465–495, DM 505; BA 515–555), red TRITC (EX 540/25, DM 565, BA 605/55), and blue DAPI (EX 340–380, DM 400, BA 435–485) and were stained with auramine O (Heslop-Harrison 1977). The observations were recorded using a Nikon 90i fluorescence microscope equipped with digital camera (Nikon Fi1) and NIS-Elements Br 2 software.

Hand-cut sections from fresh flowers as well as pedicels and bracts were viewed in water and after application of the histochemical assays in order to determine the structure and content of stigma and nectary cells as well as trichome cells. The following histochemical assays were employed: IKI solution (Johansen 1940) for starch detection, NADI reagent (David and Carde 1964) for terpenes, Sudan IV (Pearse 1985; Brundrett et al. 1991) for stain lipids, Nile Blue (Jensen 1962) for neutral and acidic lipids, ruthenium red (Johansen 1940; Jensen 1962) for presence of mucilage, neutral red (Conn 1977; Lulai and Morgan 1992) for lipophilic structures and lipids, and potassium bichromate (Gabe 1968) to detect tannins. Whilst using all the histochemical methods, standard control procedures suggested by the respective authors were simultaneously applied. All sections were examined using a Nikon Eclipse E200 light microscope (Nikon, Japan), and the measurements of the length of each type of the grandular trichomes (*n* = 20) were taken.

## Results

### Micromorphology of *Viburnum opulus* flowers

**Fig. 1:**
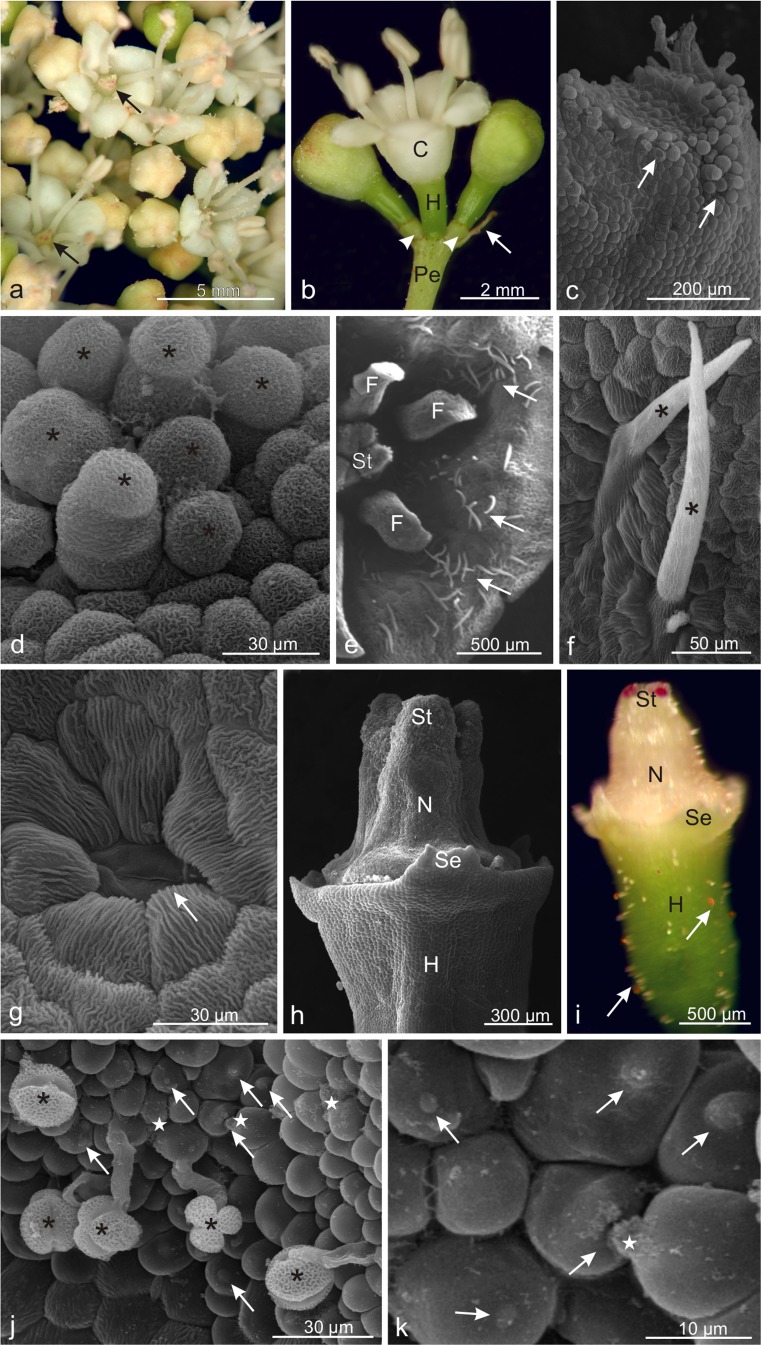
Micromorphology of *Viburnum opulus* flowers. **a** Fragment of an umbel-like inflorescence with hermaphroditic flowers with red stigmas (*arrows*). **b** Flower and two buds on short pedicels (*arrowheads*) with a visible bract (*arrow*). **c** Fragment of a corolla lobe with protruding papillae (*arrows*). **d** Papillae (*asterisks*) on the surface of the apical part of the petal lobe. **e** Top view of a fragment of the flower with simple non-glandular trichomes (*arrows*) on the adaxial surface of the petals. **f** Non-glandular trichomes on a corolla petal (*asterisks*). **g** Cells of adaxial epidermis of a petal with a stoma (*arrow*). **h, i** Fragments of the hypanthium with the style, three-lobed stigma, and a nectary located around the style. Note red stigma and inconspicuous glandular trichomes (*arrows*) on the hypanthium surface (**i**). **j**, **k** Papillae on the stigma surface with light areas (*arrows*), pollen grains (*asterisks*), and a flocculent secretion (*stars*); *C* corolla, *F* filament of stamen, *H* hypanthium, *Pe* peduncle, *St* stigma, *N* nectary

The pentamerous *V. opulus* flowers gathered in corymb-like inflorescences were differentiated into marginal sterile, large, white flowers equipped with a pistil having smaller or ruptured stigmas and 4 underdeveloped stamens as well as highly numerous, inconspicuous, inner creamy-white hermaphroditic flowers with an unpleasant odour and 5 stamens equipped with long filaments and bright yellow anthers (Fig. 1a, b). On the first day of anthesis, immediately after opening of fertile flowers, rapid rupture of anthers and tiny nectar droplets on the nectary surface were observed. In their apical part, fused petals formed lobes with protruding or elongated papillae at the margins (Fig. 1c, d). The adaxial surface of the petals had uniseriate, unicellular, sharp-pointed non-glandular trichomes covered by a striated cuticle (Fig. 1e, f) and slightly sunk stomata (Fig. 1g). The epidermis cells of the petals were characterised by a puzzle-like shape and presence of deep cuticular striae (Fig. 1f, g). The cylindrical creamy-colour style and the base of the inferior ovary were surrounded by a similarly coloured nectary (Fig. 1h, i). The style had a three-lobed stigma, which was stained red by anthocyanins and was formed of numerous protruding papillae covered by a smooth cuticle (Table 2, Fig. 1a, h–k). At the apex of the papillae, there were slightly convex, oval, brighter areas, probably indicating the sites of cuticle rupture and release of a flocculent secretion on the surface of some papillae (Fig. 1j, k). On the surface of the stigma, there were tricolporate, round pollen grains, sometimes germinating, which exhibited reticulate ornamentation. The stigmas of fresh flowers were stained by Sudan IV, rhutenium red, and neutral red (not shown). The highly elongated and flattened hypanthium was characterised by the presence of short glandular trichomes, which usually had light yellow and orange-ginger-brown heads (Fig. 1i). This type of trichomes was more densely distributed at the hypanthium base and on the adjacent flower pedicel.

**Table 2:** Comparison of the microstructure of *Viburnum opulus* and *V. lantana* flowers

Characteristics	Mean ± SD	
	*V. opulus*	*V. lantana*
Length of the style (μm) *n* = 10	693.7 ± 41.3	1098.0 ± 74.2
Height of the stigma (μm) *n* = 10	232.6 ± 15.7	30.9 ± 5.6
Diameter of the stigma (μm) *n* = 10	425.27 ± 21.4	952.3 ± 38.7
Type of stigma	Wet stigmas	
Colour of stigma	Red	Yellow-green
Diameter of the style (nectary) under the stigma (μm) *n* = 10	512.2 ± 13.7	950.3 ± 19.9
Diameter of the style (nectary) at the base (μm) *n* = 10	602.0 ± 12.5	1325.1 ± 35.1
Height of the nectary (μm) *n* = 10	448.9 ± 21.4	492.3 ± 17.5
Area of the nectary (mm^2^) *n* = 10	0.6 ± 0.05	1.8 ± 0.2
Location of the nectarostomata	At the level of epidermis cells	Below the level of epidermis cells
Number of nectarostomata in mm^2^ area of the nectary epidermis *n* = 5	540 ± 17	652 ± 12
Length of nectarostomata (μm) *n* = 20	24.9 ± 7.2	20.4 ± 5.7
Number of the glandular parenchyma layers *n* = 10	6 ± 1	9 ± 1
Tannin deposits	Few, tiny	Numerous, large
Non-glandular trichomes	Simple, on corolla petals	Stellate, on bracts and floral peduncles and pedicels
Glandular trichomes	On hypanthia, floral pedicels and peduncles: capitate, peltate, digitiform	On hypanthia, bracts, floral pedicels and peduncles: capitate
Composition of glandular trichome secretions	Lipids, mucilage, and tannins	

### Microstructure of *Viburnum. opulus* trichomes

**Fig. 2:**
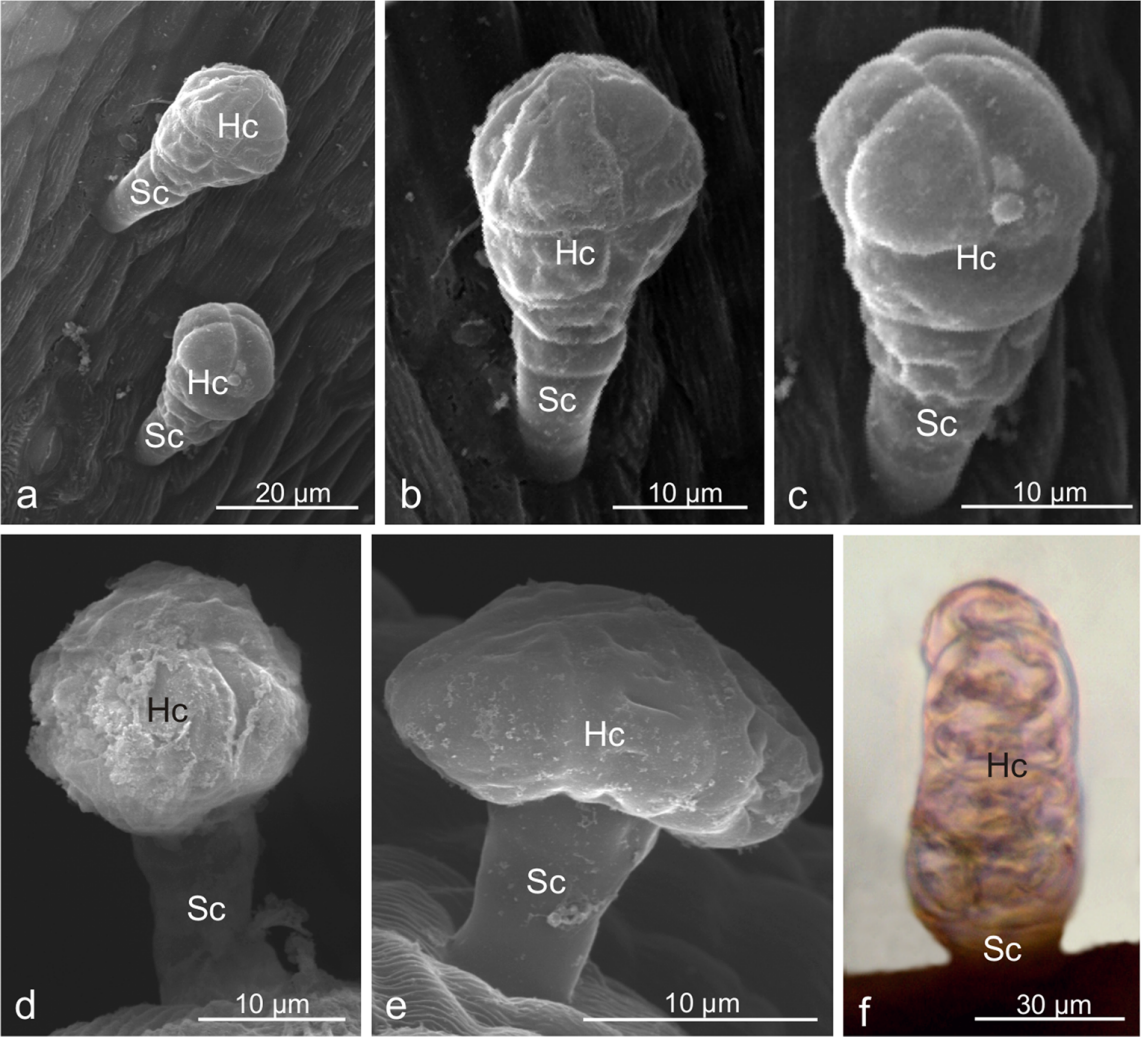
Microstructure of glandular trichomes of *Viburnum opulus*. **a-c** Capitate clavate trichomes with a multicellular several-layer head. **d** Capitate trichomes with a bicellular head. **e** Peltate trichomes with a flattened head formed by several cells arranged in a circle. **f** Digitiform trichome; *Hc* head cells, *Sc* stalk cells

The glandular trichomes in *V. opulus* exhibited a varied size and structure (Table 2, Fig. 2): *i*- the most common capitate trichomes with an average length of ca. 80 μm formed of a 2–5-celled stalk and a multicellular, clavate secretory head formed of several multicellular cell layers. The top layer was usually formed of 4 cells (Fig. 2a-c); *ii*- capitate trichomes with a 1–2-celled stalk and bicellular spherical head and an average length of 87 μm (Fig. 2d); *iii-* 62-μm-long peltate trichomes most common on the hypanthium composed of a 1–3-celled stalk and a large, flattened head formed by several secretory cells (Fig. 2e); *iv*- the least frequent ca. 100-μm-long digitiform trichomes with a 1–3-celled stalk and a several-layer head comprising one cell in each layer (Fig. 2f). The head cells were arranged linearly and had similar diameters; frequently, it was difficult to distinguish between the head and the stalk cells. In general, the trichomes present on the hypanthium were by over 10 % lower than the trichomes located on the pedicels. Dried secretion was noted on the heads of some trichomes (Fig. 2b–e). No glandular trichomes were found on the bracts.

### Microstructure of *Viburnum opulus* nectary

**Fig. 3:**
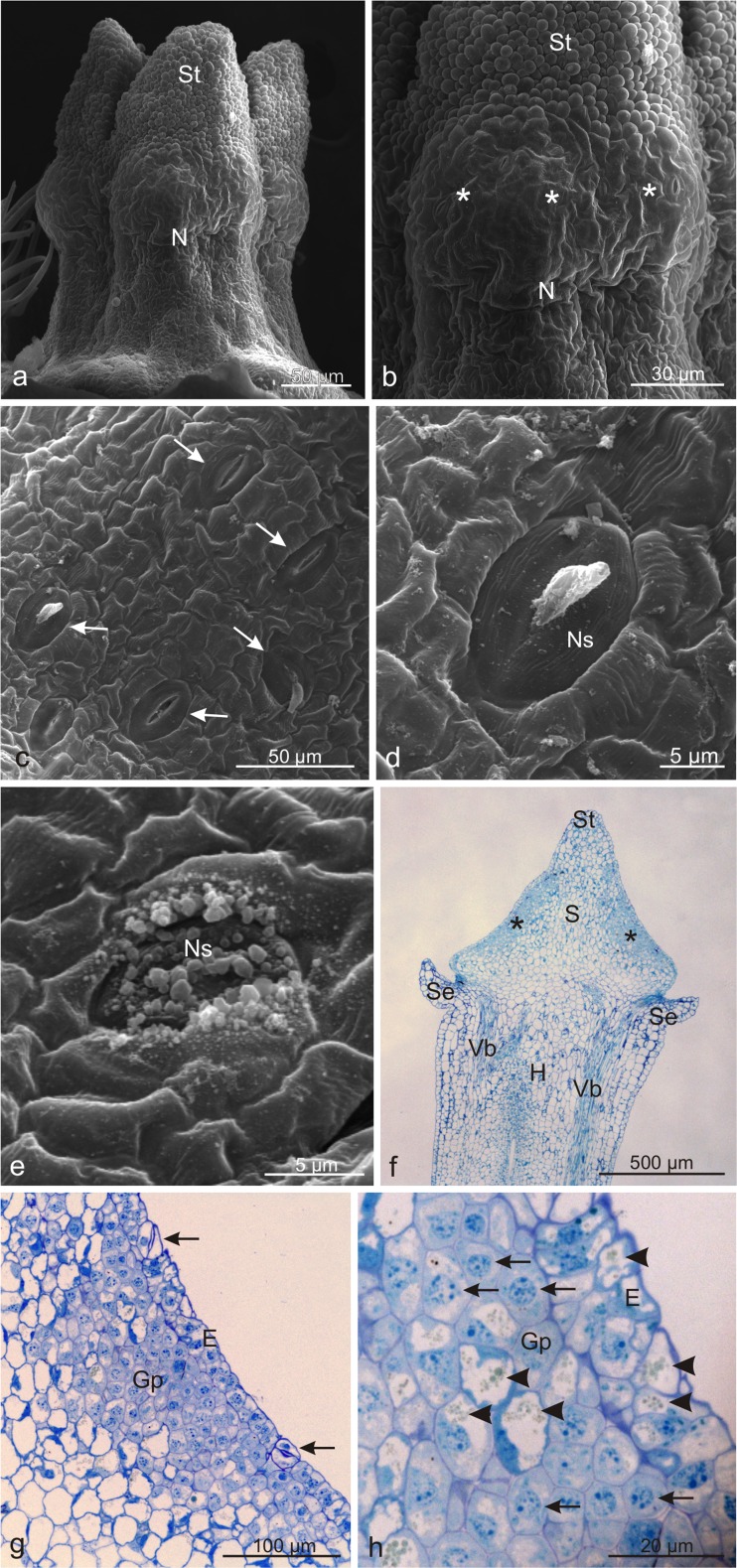
Microstructure of *Viburnum opulus* nectary. **a** The style with the stigma and a surrounding nectary. **b** A visible heterogeneous nectary surface. *Asterisks* indicate the protruding part of the nectary. **c** Fragment of the nectary surface with stomata (*arrows*). **d, e** nectarostomata with a secretion visible inside the porus (**d**) and on the stoma surface (**e**). **f** Longitudinal section of the hypanthium and style with a nectary (*asterisks*). **g** Longitudinal section of the nectary with glandular parenchyma and stomata (*arrows*) in the epidermis. **h** Cells of the nectary epidermis and glandular parenchyma with cell nuclei (*arrows*) and tannin deposits in the vacuoles (*arrowheads*); *Se* sepals, *S* style, *St* stigma, *N* nectary, *Ns* nectarostomata, *H* hypanthium, *E* epidermis, *Gp* grandular parenchyma, *Vb* vascular bundles

The nectary gland in the flowers of *V. opulus* was located around the style and bordered the stigma on one side and the hypanthium on the other side (Table 2, Fig. 3a). The part adjacent to the stigma was slightly protruding and its epidermal cells had an irregular shape; in turn, the part bordering the hypanthium was composed of smaller epidermal cells with varied shapes (Fig. 3a–c). Between the epidermal cells, there were densely arranged anomocytic nectarostomata (Table 2, Fig. 3c–e). Open stomata with a visible secretion in their pores or numerous granules on the surface were observed most frequently (Fig. 3d, e). The stomata on the convex part of the gland were arranged chaotically, whereas the direction of the long axis of the stomata located on the hypanthium-bordering part was most often consistent with the vertical axis of the style (Fig. 3c).

Longitudinal sections revealed that the nectary gland formed a thick layer of intensively stained, compactly arranged, tiny, polygonal cells with a dense cytoplasm and a varying degree of vacuolation (Fig. 3f–h). The nectary was typically built of single-layer epidermis and 5–7-layer glandular parenchyma (Fig. 3g, h). In the epidermis, whose cells were characterised by thin outer walls, there were stomata (Fig. 3g) with cells containing starch grains stained by IKI. The cells of the epidermis and glandular parenchyma exhibited large cell nuclei with nucleoli. Vacuoles of many glandular parenchyma cells contained different-size green-blue oval deposits of tannin compounds (Fig. 3g, h). The nectary was not equipped with elements of the vascular tissue. However, near the gland, there were vascular bundles of the sepals and style (Fig. 3f).

### Micromorphology of *Viburnum lantana* flowers

**Fig 4:**
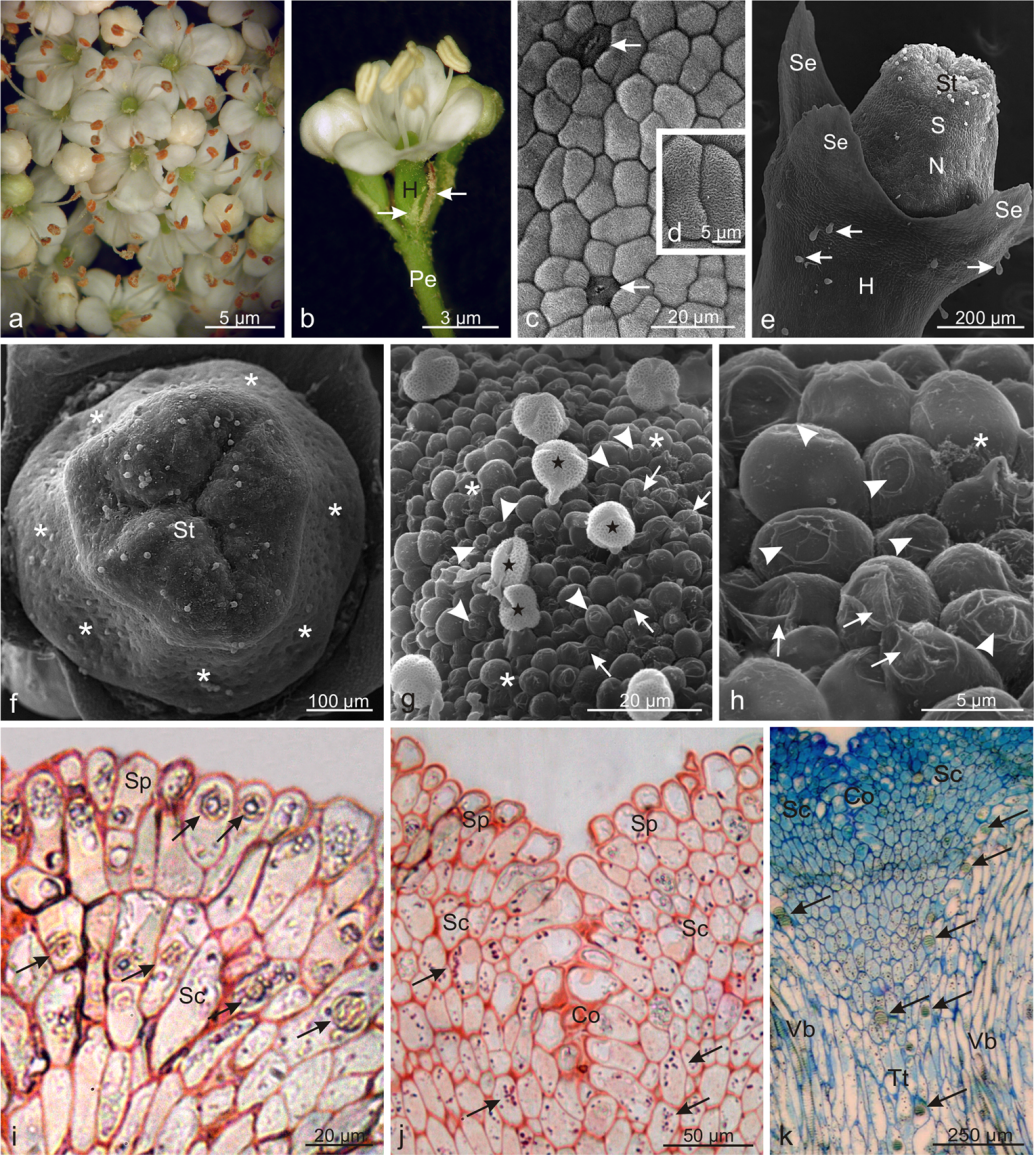
Micromorphology of *Viburnum lantana* flowers. **a** Fertile, hermaphroditic flowers in an umbel-like inflorescence in the anthesis stage. **b** Freshly opened flower with two buds with visible bracts (*arrows*). **c, d** Cells of the adaxial epidermis of a petal with stomata (*arrows*) and massive cuticular striae (**d**). **e** Visible the style, stigma, and nectary located around the style. Note grandular trichomes (*arrows*) on the hypanthium surface. **f** Top view of the surface of the three-lobed stigma with pollen grains and the surface of a nectary (*asterisks*) with numerous stomata. **g, h** Papillae on the stigma surface with visible regions of a strongly wrinkled cuticle (*arrows*) and oval, flat or concave areas surrounded by the cuticle forming a characteristic ring (*arrowheads*). Note the pollen grains (*stars*) and the flocculent secretion (*asterisks*). **i** Visible stigmatic papillae and outer stigmatic cells stained by PAS with visible tannin deposits (*arrows*). **j** Numerous PAS-stained starch grains (*arrows*) located in stigmatic cells adjacent to the stigmatic *compitum*. **k** Stigmatic cells and stylar transmitting tissue formed by several bands of elongated cells with tannin deposits (*arrows)*; *Pe* peduncle, *Se* sepal, *H* hypanthium, *St* stigma, *N* nectary, *Sp* stigmatic papillae, *Sc* stigmatic cells, *Co* stigmatic *compitum*, *Tt* transmitting tissue, *Vb* vascular bundles

The umbel-like inflorescences of *V. lantana* were mainly built of inconspicuous, fertile, hermaphroditic, pentamerous flowers characterised by a disagreeable odour (Fig. 4a, b). On the first day after anthesis, rapid nectar secretion on the style surface and anther dehiscence were observed. During pollen release, the anthers changed their colour from light yellow to orange (Fig. 4a, b). The margins of corolla petals had elongated, protruding papillae, similar to those observed in *V. opulus* (not shown). The polygonal cells of the adaxial epidermis of corolla petals were characterised by a strongly striated cuticle and the presence of stomata (Fig. 4c, d). The style of the pistil in the shape of a truncated cone was equipped with a nectary gland surrounding it (Fig. 4e, f). The style in *V. lantana* pistils was twice as wide at the base and longer by half than that in *V. opulus* (Table 2, Fig. 4e, f). Similarly, the diameter of the stigma in *V. lantana* was over twofold greater than that in *V. opulus*. The three-lobed stigma was not high and had an undulated surface formed of numerous, unicellular, spherical, different-size papillae (Fig. 4f–h). At the apex of many of the papillae, there were oval, flat, or sometimes hollow areas delimited by elevated cuticle rings or areas with a strongly wrinkled cuticle, through which the secretion observed on the surface of some papillae was probably released (Fig. 4g, h). The longitudinal sections showed that the intensely PAS-stained papillar cells and the elongated inner stigmatic cells forming the stigma contained oval deposits of tannin compounds and starch grains, which were particularly abundant in the cells of the stigmatic zone adjacent to the *compitum* and forming the stylar transmitting tissue (Fig. 4i, j). The transmitting tissue formed an oblong band of several strands of elongated cells accompanied by vascular bundles on both sides (Fig. 4k). Large tannin deposits were present in many cells of the transmitting tissue. The inner stigmatic cells were continuous with the stylar transmitting tissue, exhibiting an overall funnel-shaped arrangement, which was wider in the stigma and basipetally narrower in the style (Fig. 4k).

### Microstructure of *Viburnum lantana* trichomes

**Fig. 5:**
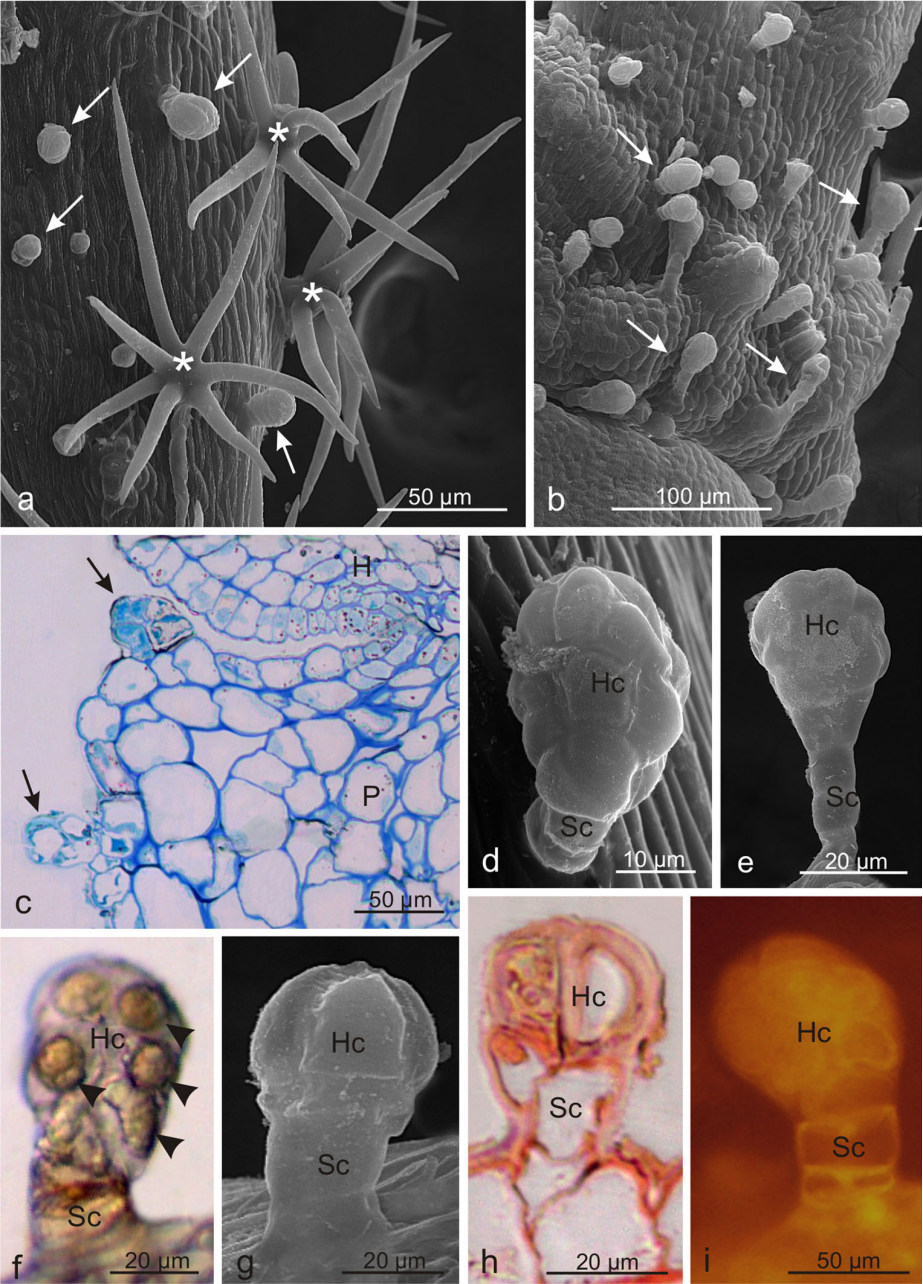
Microstructure of trichomes of *Viburnum lantana*. **a** Stellate non-glandular (*asterisks*) and glandular trichomes (*arrows*) on the peduncle surface. **b** Numerous glandular trichomes (*arrows*) on the surface of the pedicel. **c** Fragment of a longitudinal section of the hypanthium and a pedicel with glandular trichomes (*arrows*) stained by PAS. **d, e** Capitate clavate trichomes with a multicellular secretory head. **f** Fresh clavate trichome with visible oval deposits (*arrowheads*) in the head cells. **g** Capitate trichome with an oval, multicellular head. **h** Longitudinal section of a PAS-stained capitate trichome. **i** Visible autofluorescence of the cuticle and secreted materials inside secretory cells; *H* hypanthium, *P* pedicel, *Hc* head cells, *Sc* stalk cells

The grey-beige bracts and inflorescence pedicels and peduncles in *V. lantana* flowers were covered densely by stellate non-glandular trichomes, between which short glandular trichomes (Fig. 5a–c), usually with orange, red-brown, or brown heads, were observed. Glandular trichomes were also visible on the hypanthium surface and sepals. They had a varied height and structure. The longest (average length ca. 87 μm) and the most abundant were the capitate clavate trichomes with a 2–3-cell stalk and a multicellular head formed of 2 or 3 layers of secretory cells. In each layer, there were several secretory cells, often with orange-brown content or with large spherical deposits in fresh trichomes (Fig. 5d–f). The capitate trichomes with an approximate length of 73 μm, 1- or 2-celled stalk, and a 1-or 2-layered head composed of 2 or 4 cells in each layer were less numerous (Fig. 5g, h). In fresh trichomes, this type of secretory cells often exhibited stained content. Furthermore, the cuticle on the surface of both types of trichomes and the content of the secretory cells showed intensive autofluorescence (Fig. 5i).

**Fig. 6:**
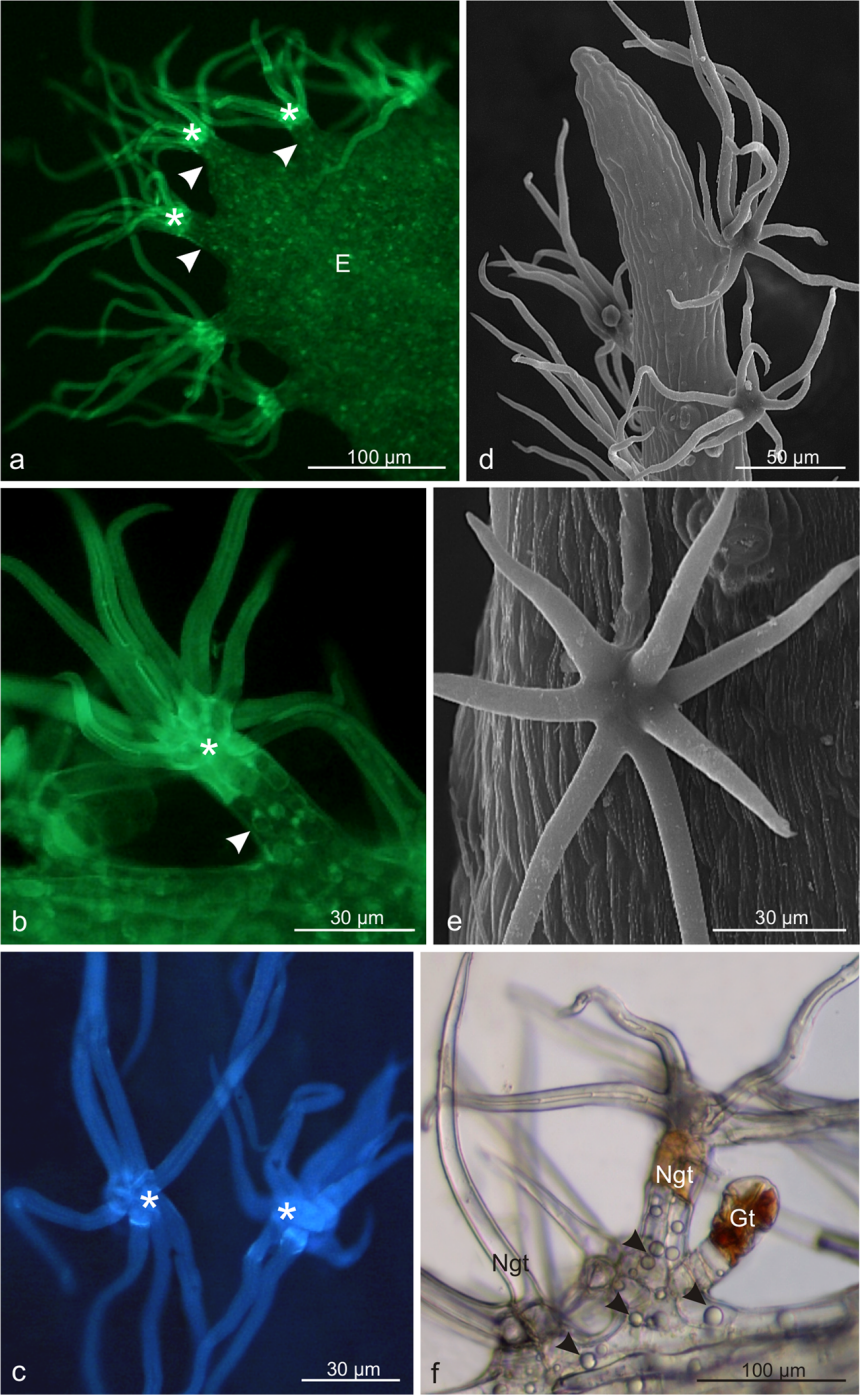
Microstructure of non-glandular trichomes of *Viburnum lantana*. **a-c** Stellate trichomes on the bract surface viewed under a fluorescence microscope. Note the intense autofluorescence of the arms and stalk cells *(asterisks)*. Autofluorescence is also exhibited by the lipid droplets contained in the cells of epidermis protrusions (*arrowheads*) and in other cells of the bract epidermis (**a, b**). **a, b** Lateral view. **c** Top view. **d, e** Trichomes viewed in SEM. **f** Visible different type of trichomes and lipid droplets (*arrowheads*) in the cells of the bract epidermis; *E* epidermis, *Sc* stalk cells, *Gt* glandular trichome, *Ngt* non-glandular trichomes

The stellate non-glandular trichomes were located on the protrusions of the epidermis cells and were formed by 2, 6, 8, or 10 arms and a multicellular stalk, which was brown-orange in the fresh trichomes (Fig. 6a–f). The cells of the stalk and the junction of the trichome arms exhibited intensive autofluorescence in the presence of all the filters used (Fig. 6a–c). In turn, the cells of the epidermis protrusion and many epidermis cells of the bracts and inflorescence pedicels were characterised by the presence of tannins (not shown) and numerous, large lipid droplets, which were visible in the fluorescence microscope and in fresh trichomes (Fig. 6a, b, f).

**Fig. 7:**
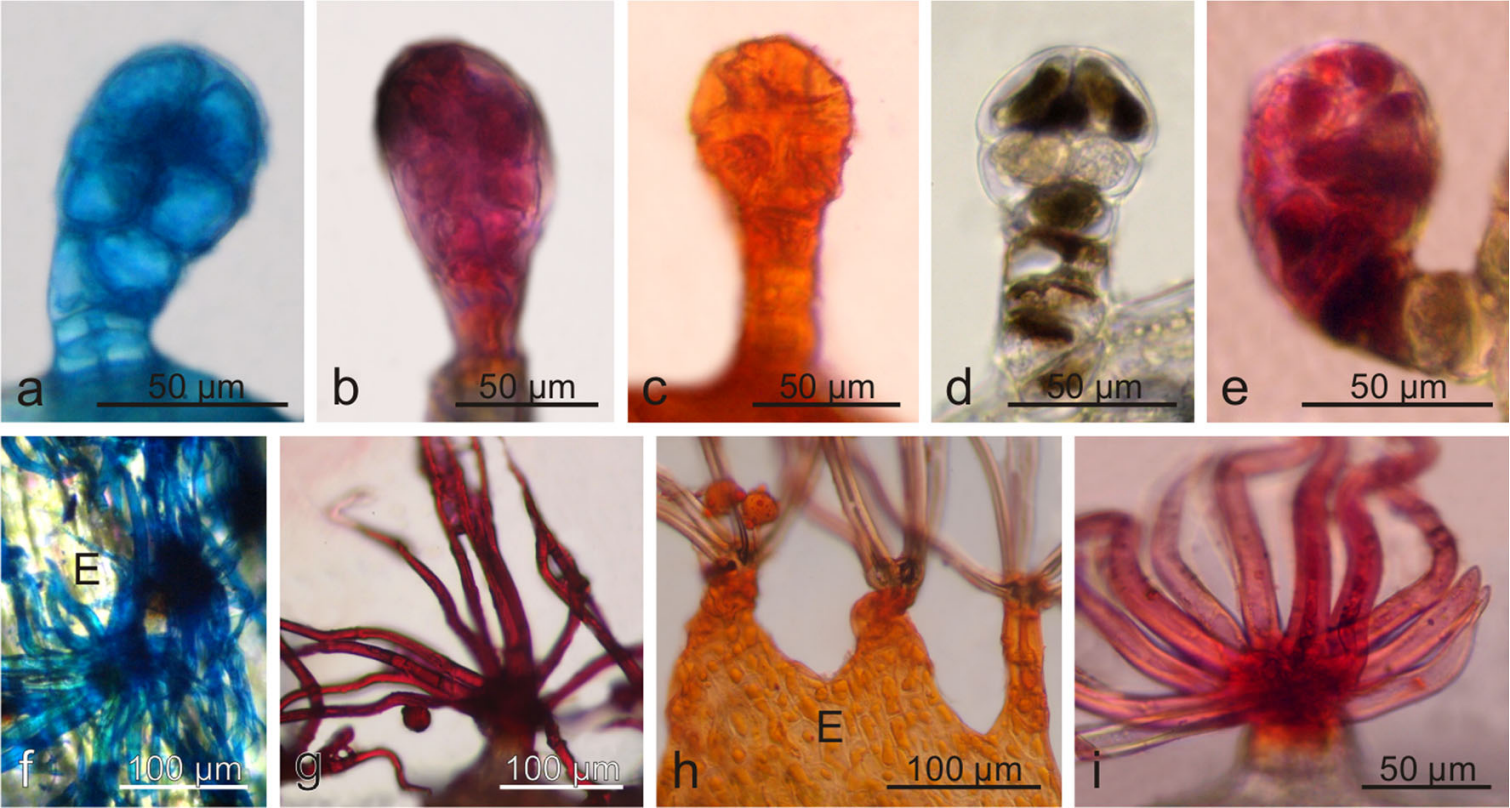
Histochemistry of trichomes of *V. opulus* (**a-c**) and *V. lantana* (**d-i**). **a-e** glandular trichomes. **f-i** non-glandular trichomes. **a, f** after Nile blue staining. **b, g** after neutral red staining. **c, h** after Sudan IV staining. Note lipid droplets in the epidermis of the bract (**h**). **d** after dichromate potassium staining. **e, i** after ruthenium red staining; *E* epidermis of bract

In both *Viburnum* species, the histochemical assays used revealed the presence of lipids, mucilage, and tannins (Fig. 7a–e), in the cells of the secretory trichomes, and mucilage and lipids in the non-glandular trichomes in *V. lantana* (Fig. 7f–i). Treatments with Nadi reagent for terpenes proved negative.

### Microstructure of *Viburnum lantana* nectaries

**Fig. 8:**
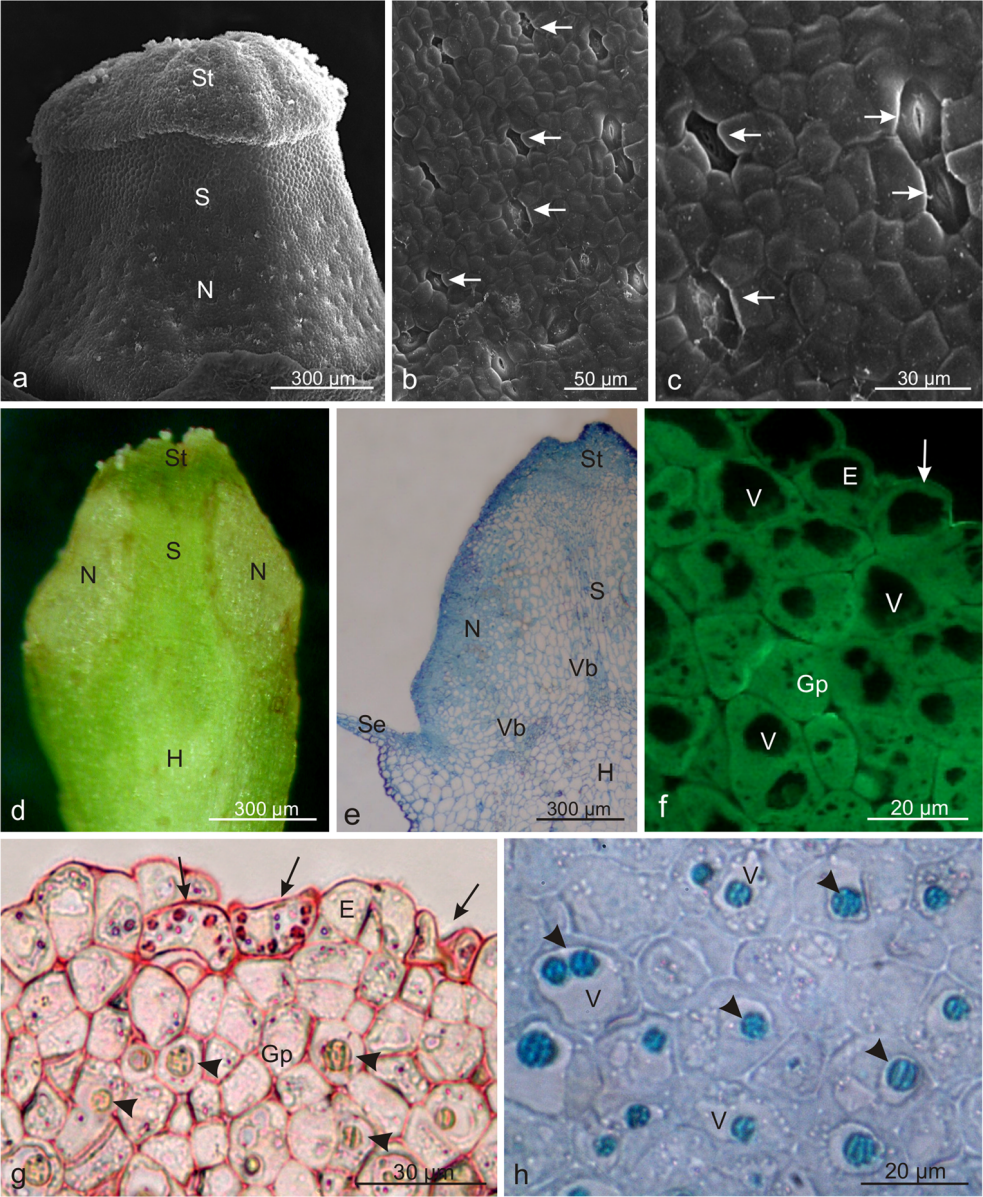
Microstructure of *Viburnum lantana* nectary. **a** Visible style with the stigma and surrounding nectary. **b** Fragment of the nectary surface with numerous concave nectarostomata (*arrows*). **c** Concave nectarostomata on the nectary surface (*arrows*). **d** Longitudinal section of a fresh hypanthium and a style with a nectary. **e** Longitudinal section of the style with a nectary. Note the intense staining of the nectary tissues. **f** Nectary epidermis cells with a very thin, almost invisible cuticle (*arrows*) on their surface, which stains very slightly with auramine O. **g** PAS-stained starch grains in the nectarostomata (*arrows*) and tannin deposits (*arrowheads*) in the vacuoles of glandular parenchyma cells **h** Large tannin deposits (*arrowheads*) in the vacuoles of glandular parenchyma cells; *S* style, *St* stigma, *N* nectary, *H* hypanthium, *E* epidermis, *Gp* grandular parenchyma, *Vb* vascular bundles, *V* vacuoles

As in *V. opulus*, the nectary in *V. lantana* flowers was located around the style, although not along its entire length (Fig. 8a). Between the nectary and the stigma, there was a zone of smaller cells covered by a striated cuticle and devoid of stomata (Fig. 8a). The surface area of the nectary in this species was threefold greater than that in the *V. opulus* (Table 2). More numerous nectarostomata, smaller than those in *V. opulus,* which exhibited a varied degree of porus opening and were usually located below the level of other epidermis cells, were observed on the nectary surface (Table 2, Fig. 8b, c). The longitudinal axis of the nectarostomata was most frequently directed in along the vertical axis of the style.

The nectary tissue observed in fresh sections formed a substantially thick layer with a lighter colour than the adjacent style tissues (Fig. 8d). The semi-thin sections revealed that the nectary gland was built of intensively stained, compactly arranged cells forming an epidermis with numerous, slightly concave stomata and an 8–10-layer glandular parenchyma (Table 2, Fig. 8e–h). The epidermis cells were characterised by thin outer cell walls and an almost invisible cuticle, which was stained very slightly with auramine O, and by the presence of different-size vacuoles (Fig. 8f, g). The glandular parenchyma was formed of small, various-size cells with a dense cytoplasm and a varied vacuolation degree (Fig. 8f–h). The vacuoles of the glandular parenchyma contained large, oval deposits of tannin compounds stained dirty yellow in PAS or purple and turquoise in methylene blue (Fig. 8g, h). Starch grains were located in the nectarostomatal cells and were rarely observed in the glandular parenchyma cells (Fig. 8g). As in the *V. opulus* nectaries, the secretory parenchyma of *V. lantana* did not contain any elements of vascular tissue, whereas vascular bundles of the style and hypanthium were present in close proximity, in the stylar cortex layer (Fig. 8e).

## Discussion

In the case of entomophilous plants, successful pollination and, consequently, fertilisation and seed and fruit development are dependent on insect pollinator visits. The presence and efficiency of pollinators is related to the attractiveness of flowers, and especially the presence of the signal and food attractants. The homogamous *Viburnum opulus* and *V. lantana* flowers, which are pollinated by a number of insect groups, were characterised by a specific, disagreeable odour. At the petal margins in both species, there were elongated papillae, which were larger than others and were probably the site of odour emission. Dötterl et al. (2006) found that lilac aldehyde, which is of special interest to pollinators, was one of the compounds in floral scents of *Viburnum opulus* emitted in high amounts. A similar insect-attractant function is served by the ring of large sterile flowers surrounding of the inflorescences in *V. opulus* and other *Viburnum* species, e.g. *V. furcatum*, *V. plicatum*, *V macrocephalum*, or *V. trilobum* (Donoghue 1980; Jin et al. 2010).

The stigmas in the analysed *Viburnum* species differed in their colour and size, but their structure was similar. Donoghue (1980) claim that the varied colouration of stigmas in different *Viburnum* species is one of their taxonomic traits and their red colour may be more attractive to specific groups of insect pollinators, e.g. butterflies and moths. Moreover, Yang et al. (2015) suppose that red stigmas may operate more effectively during flowering at lower temperatures. At the apex of many papillae forming *V. opulus* and *V. lanatana* stigmas, there were characteristic protruding areas or round traces surrounded by an elevated cuticle, which indicated the route of the outflow of the stigmatic secretion. This type of stigmas as in *Viburnum*, with their the surface of which typically bears a liquid secretion during the receptivity period, are called “wet stigmas”, in contrast to “dry stigmas”, which have intact surface cells that typically protrude as papillae and are covered by a primary cell wall, a waxy cuticle, and a proteinaceous pellicle (Heslop-Harrison 1977, 1981). As reported by Heslop-Harrison and Shivanna (1977) and Cronquist (1981), “wet stigmas” are also present in *Sambucus*, whereas “dry stigmas” were observed in other Adoxaceae species. Based on the various histochemical tests used in this study, it was found that the extracellular components of the *Viburnum* stigmatic surface were heterogeneous and included carbohydrates (mucilage), lipids, and tannins, which contribute to rapid pollen germination, whereas *V. opulus* also exhibited the presence of anthocyanins. Furthermore, Wolters-Arts et al. (1998) have found that lipid components seem to be essential for pollen tube penetration in the stigma and ulterior growth through the style. In turn, in the reproduction process, carbohydrates are a source of nutrients indispensable for pollen tube growth (Herrero and Dickinson 1979) or for the development of the ovary and ovules (Arbeloa and Herrero 1991).

The floral nectaries located around the style in the analysed *Viburnum* flowers are easily accessible for insect pollinators. Moreover, they represent the persistent nectary type (nectaria persistentia), since their dried parts remain at the time of fruit ripening (Smets 1986). Fahn (1952) classified this type of nectaries located around the base of the style as “style nectaries”. Other researchers report that the floral nectaries in the genus *Viburnum* form a disc-like nectary on the top of the gynoecium and represent “gynoecial nectaries” (Erbar 1994; Erbar and Leins 2010; Tank and Donoghue 2010). This type of nectaries are not present in other representatives of the family Adoxaceae, which exhibit trachomatous nectaries located at corolla lobe bases (*Adoxa*, *Sinadoxa*, *Tetradoxa*) or whole sterile flowers in the inflorescence were converted into nectaries (some species of *Sambucus*) (Vogel 1997; Donoghue et al. 2003). Some *Viburnum* and *Sambucus* species have extrafloral nectaries as well; they are mainly attractive to ants, which may protect plants against herbivore attacks (Fahn 1987; Weber et al. 2012; Clement et al. 2014).

Nectar in both species was secreted through numerous nectarostomata; their number as well as the nectary surface area and the number of glandular tissue layers were greater in *V. lantana*, which suggests that the flowers of the former species produce bigger amounts of nectar. Many researchers believe that the weight of secreted nectar is strictly correlated with the size (thickness and secretory surface) of the nectary (Orosz Kovács et al. 1990; Scheidné Nagy Tóth and Orosz Kovács 2001) and the number of nectarostomata (Davis and Gunning 1991; Weryszko-Chmielewska et al. 1996). Moreover, the great number of nectarostomata in the analysed *Viburnum* species implies an intensive and short-term nectar outflow; similarly, the very thin and smooth cuticle on the nectary surface promotes rapid drying of nectar and, hence, short-term supply thereof. In turn, the small sizes of the nectaries in the inconspicuous *V. opulus* and *V. lantana* flowers suggest production of small amounts of nectar. As suggested by López and Galetto (2002), in the case of a great number of simultaneously blooming flowers, e.g. in *Viburnum*, the quantity of nectar cannot be high in order to prevent overloading the plants with the cost of production thereof. These relationships have also been reported by some researchers, which confirms the fact that *Viburnum* flowers produce low amounts of weak nectar for a short time; yet, they are attractive to many different groups of pollinators, which ensures effective cross-pollination (Donoghue 1980; Kollmann and Grubb 2002; Jin et al. 2010). Furthermore, Denisow et al. (2015) report that even if a single flower of a species produces small amounts of nectar but the number of plant flowers is high and/or the plants form a dense canopy, the species can be an attractive source of nectar for pollinators. The composition of nectar and pollen productivity in different *Viburnum* species may also determine the attractiveness of this taxon to such a great variety of pollinators. These traits have not been researched so far but they deserve further investigation. In these studies, I also found that the nectaries of the inconspicuous *Viburnum* flowers were not equipped with vascular tissue. Pre-nectar components were supplied through the vascular bundles of the adjacent organs, i.e. the style, hypanthium, and/or elements of the perianth. Similarly, no vascularisation of the nectary gland has been reported by other authors in other species producing small flowers (De Craene et al. 2014; Konarska 2015). The literature does not provide any information on the nectar production and microstructure of the floral nectaries in *Viburnum*.

On the hypanthia in both *Viburnum* species and in close proximity to the flowers, i.e. on the pedicels, peduncles and bracts, there were small-size glandular trichomes of various types: capitate (*V. opulus* and *V. lantana*), peltate, and digitiform (*V. opulus*). Moreover, stellate non-glandular trichomes covering densely the bracts, pedicels, and peduncles in *V. lantana* and simple non-glandular trichomes on adaxial surface of the petals in *V. opulus* were found. On leaves and stems of various *Viburnum* species belonging to different species and clades, many investigators have observed a similar type of glandular (Table 3) and non-glandular trichomes as well, differing in the size, number, and arrangement of trichome cells (Winkworth and Donoghue 2005; Prabhu and Ponnudurai 2011; Clement et al. 2014; Moura et al. 2015). As reported by the authors mentioned, the structure of trichomes present in different *Viburnum* species is a taxonomic trait that is important for the phylogenesis of the genus.

**Table 3:** Types of glandular trichomes present in different *Viburnum* species

Trichomes	Species or clade	Source
Capitate	*Euviburnum*	Clement et al. 2014
	*Dentata*	
	*Lobata*	
	*Mollotinus*	
	*Opulus*	
	*Oreintotinus*	
	*Solenotinus*	
	*Succotinus Tomentosa*	
	*V. lutescens*	
	*V. lantana*	Konarska this study
	*V. opulus*	
Peltate	*Lentago*	Clement and Donoghue 2011
	*V. punctatum*	Prabhu and Ponnudurai 2011
	*Coriacea*	Clement et al. 2014
	*Lentago*	
	*Punctata*	
	*Sambucina*	
	*Succotinus*	
	*V. lantana*	Konarska this study
	*V. opulus*	
Elongate	*V. clemensia*	Clement et al. 2014
	*Pseudotinus*	
	*Urceolata*	
	*Tinus*	
	*V. amplificatum*	
	*V. lutescens*	
Club-shaped	*V. erubescens*	Prabhu and Ponnudurai 2011
Rosette	*V. coriaceum*	
Digitiform	*V. opulus*	Konarska this study

The glandular trichomes in *Viburnum opulus* and *V. lantana* contained lipids, mucilage, and tannins, whereas the non-glandular trichomes exhibited the presence of lipids and mucilage. Additionally, in *V. lantana*, large lipid droplets were present in the epidermis cells of the sepals, bracts, and pedicels. In turn, tannins were contained not only in the secretory cells of the trichomes but also in the cells of the sepals, hypanthia, stigmas, and nectaries. Plant lipids constitute a large group of compounds comprising, e.g. waxes, sterols, terpenes, glycol- and phospholipids, and fatty acids, which can serve a number of functions; they may attract or guide insects, as well as constitute floral reward to pollinators (chemical attractant) (Johnson 1975; Werker 2000). The presence of tanniferous and mucilage-containing cells in the leaves, bark, and roots of various *Viburnum* species has been reported by Wilkinson (1948) and Prabhu et al. (2009a, b, 2011). Tannins may be involved in chemical defence against herbivores and pathogens by entrapping or poisoning them and appear to be a mechanism of protection of flowers, in particular their generative organs, and thus they may increase the reproductive potential of *Viburnum*. In turn, the presence of non-glandular trichomes often represents ecological adaptation to environmental conditions as a mechanical barrier protecting against increased transpiration, UV-B radiation, and extreme temperatures (Agrawal and Fishbein 2006), reduce insect movement (Kessler and Baldwin 2002) or preventing pests from oviposition (Nishida 2002). The contents of mucilage, tannins, and other secondary metabolites such as sesquiterpenes, triterpenes, phytosterols, flavonoids, and irridoid glycosides detected in roots, stems, and leaves of various *Viburnum* species are responsible for the multifarious medicinal properties of these plants, e.g. diuretic, cardiovascular stimulant, uterine sedative, antimicrobial, anti-inflammatory, antinociceptive, antispasmodic, anti-asthmatic, and astringent activities (Altun et al. 2008; Bibi et al. 2010).

The investigations have shown that the flowers of *Viburnum lantana* and *V. opulus* are characterised by many adaptations to insect pollination. They exhibit numerous homologous traits related to the micromorphology of the petals and stigmas, the distribution and anatomy of nectaries, the mode of nectar secretion, and the composition of stigmatic and trichome secretions. Both *Viburnum* species also differed in a number of quantitative and qualitative charactristics of the microstructure of pistils, nectaries, and glandular and non-glandular trichomes. It was found that the location and type of *Viburnum* nectaries were substantially unlike from the model described in other representatives of Adoxaceae. Given the common occurrence of many *Viburnum* species as well as the great similarity between them and hybridisation potential, the original comparative investigations of the structure of the stigmas, nectaries, and trichomes in *Viburnum opulus* and *V. lantana* provide better understanding of the phylogenesis and taxonomy of the genus *Viburnum*. Furthermore, the analyses of the microstructure floral nectaries carried for the first time are a valuable complement to the knowledge of the floral and pollination ecology. The secretory product of the glandular trichomes of different *Viburnum* should be further investigated in order to identify all bioactive substances with medicinal properties contained therein.
